# Fungi in Thailand: A Case Study of the Efficacy of an ITS Barcode for Automatically Identifying Species within the Annulohypoxylon and Hypoxylon Genera

**DOI:** 10.1371/journal.pone.0054529

**Published:** 2013-02-04

**Authors:** Nuttika Suwannasai, María P. Martín, Cherdchai Phosri, Prakitsin Sihanonth, Anthony J. S. Whalley, John L. Spouge

**Affiliations:** 1 Department of Biology, Faculty of Science, Srinakharinwirot University, Bangkok, Thailand; 2 Department of Mycology, Real Jardín Botánico-CSIC, Plaza de Murillo 2, Madrid, Spain; 3 Microbiology Programme, Faculty of Science and Technology, Pibulsongkram Rajabhat University, Phitsanulok, Thailand; 4 Department of Microbiology, Faculty of Science, Chulalongkorn University, Bangkok, Thailand; 5 School of Pharmacy and Biomolecular Sciences, Liverpool John Moores University, Liverpool, United Kingdom; 6 National Center for Biotechnology Information, National Library of Medicine, Bethesda, Maryland, United States of America; Soonchunhyang University, Republic of Korea

## Abstract

Thailand, a part of the Indo-Burma biodiversity hotspot, has many endemic animals and plants. Some of its fungal species are difficult to recognize and separate, complicating assessments of biodiversity. We assessed species diversity within the fungal genera *Annulohypoxylon* and *Hypoxylon*, which produce biologically active and potentially therapeutic compounds, by applying classical taxonomic methods to 552 teleomorphs collected from across Thailand. Using probability of correct identification (PCI), we also assessed the efficacy of automated species identification with a fungal barcode marker, ITS, in the model system of *Annulohypoxylon* and *Hypoxylon*. The 552 teleomorphs yielded 137 ITS sequences; in addition, we examined 128 GenBank ITS sequences, to assess biases in evaluating a DNA barcode with GenBank data. The use of multiple sequence alignment in a barcode database like BOLD raises some concerns about non-protein barcode markers like ITS, so we also compared species identification using different alignment methods. Our results suggest the following. (1) Multiple sequence alignment of ITS sequences is competitive with pairwise alignment when identifying species, so BOLD should be able to preserve its present bioinformatics workflow for species identification for ITS, and possibly therefore with at least some other non-protein barcode markers. (2) Automated species identification is insensitive to a specific choice of evolutionary distance, contributing to resolution of a current debate in DNA barcoding. (3) Statistical methods are available to address, at least partially, the possibility of expert misidentification of species. Phylogenetic trees discovered a cryptic species and strongly supported monophyletic clades for many *Annulohypoxylon* and *Hypoxylon* species, suggesting that ITS can contribute usefully to a barcode for these fungi. The PCIs here, derived solely from ITS, suggest that a fungal barcode will require secondary markers in *Annulohypoxylon* and *Hypoxylon*, however. The URL http://tinyurl.com/spouge-barcode contains computer programs and other supplementary material relevant to this article.

## Introduction

Reliable species identification is fundamental to assessing biodiversity, geographical variation, and environmental changes, as well as to discovering novel drugs and many other experimental enterprises. Species identification in fungi is particularly challenging, because of their ephemeral nature. Moreover, fungal diversity in the tropics is particularly difficult to assess, because both taxonomic specialists and a complete understanding of the reliability of many taxonomic characters are lacking [1]. Based on our extended studies of fungi in Thailand, we assess the geographical distribution of the Xylariaceae, a family of the Ascomycota. The Ascomycota are less ephemeral than the fleshy fungi of the Basidiomycota, so the estimates of fungal diversity based on them are more robust than estimates based on Basidiomycota. The delimitation of Xylariaceae from other related pyrenomycete families has always been problematic [2], [3], so we also improved protocols for species identification within them [3]–[7].

Thailand has a rich diversity of Xylariaceae [8], containing many unknown species [3], along with 23 out of the 74 genera accepted in a standard reference [9]. Within the Xylariaceae, the genera *Annulohypoxylon* and *Hypoxylon* have received recent attention. *Annulohypoxylon* and *Hypoxylon* occur around the world, and they are very well represented in Thailand [8], [10]–[12]. Taxonomic recognition of both genera and species relies on traditional morphological characters: shape, size, color, surface features and microscopic details of the asci and ascospores. Studies using scanning electron microscopy [13]–[15] and chemical and molecular data have sharpened the boundaries of both species and genera [10], [11], [15]–[20]. Nevertheless, the separation of some closely related species remains difficult, and as in other group of fungi the number of cryptic or sibling species can be high [21]. Our inability to identify these taxa accurately impedes the assessment of species diversity and has important practical consequences for recognizing plant pathogens or discovering novel drugs.

Thailand is a tropical area in which many xylariaceous fungi are likely still unknown [3], [8], [15], [22], [23], so only a few reports on species of *Annulohypoxylon* and *Hypoxylon* are extant [10], [11], [24]. Many species reported from different localities exist only in a single collection, so without more specimens of them to display suitable distinguishing key characteristics, their taxonomy remains unhelpfully vague. A reliable, accurate, and reproducible means of identification would elucidate the diversity, distribution, and role of these taxa in nature.

DNA barcodes provide a possible technology for the requisite species identification. In its essence, a barcode is any standardized subset of DNA from a taxonomic specimen [25], [26]. To fix terminology, we use the term “marker” to connote any contiguous region of DNA (coding or non-coding).

The Internal Transcribed Spacer of nrDNA (ITS) is one of the most extensively sequenced markers in fungi (e.g. [27], [28]). Sequence analysis of ITS can provide precise information for identifying fungal species from disparate sources and for exploring their phylogenetic relationships. Although the phylogenetic trees constructed from ITS do not support the generic classification between *Annulohypoxylon* and *Hypoxylon* genera [11], [29], [30], ITS sequences have proved to be a powerful tool for identifying fungi in several problematic species complexes [11], [23], [29], [31]. Moreover, recent papers related to DNA barcoding of true fungi support the entire ITS region of the nrDNA as one of the most appropriate barcode markers for identifying fungal species in the field [32]–[35]. Accordingly, ITS has recently been selected as an official barcode marker for fungi [36].

This article combines classical taxonomic methods with sequence analysis and statistics to evaluate the suitability of ITS as a barcode marker in the specific context of the *Annulohypoxylon*-*Hypoxylon* clade, in particular, to examine whether ITS provides a good proxy for expert morphological identification and whether it aids phylogenetic study within the clade. To those aims, we systematically vary the components of the bioinformatics workflow required to exploit a marker in a barcode database like BOLD, e.g., using different alignment methods and sequence distances in our analyses below. The evaluation of a barcode requires a suitable measure of the accuracy of species identification. Barcode selection has proved problematic in some taxa, notably plants [37]–[40] and insects [41], [42], so fortunately there is now a consensus [40], [41], [43] on a suitable measure of barcode efficacy, namely the probability of correct identification (PCI) (defined in the Results section).

By comparing sequences from vouchered specimens with complete taxonomic information to sequences derived from GenBank, we explore the biases that incorrect GenBank taxonomy can introduce into scientific conclusions about barcode efficacy. By examining subsets of species where experts are likely to agree on their morphological species identification, we also evaluate possible biases introduced by incorrect morphological identification. By comparing the PCI derived from local sequence alignment (as used in the popular BLAST sequence alignment tool), to the PCI derived from alignment methods more appropriate to species identification, we explore biases that inappropriate alignment methods introduce into conclusions about barcode efficacy. Finally, before the selection of ITS as an official fungal barcode marker, all official barcode markers were protein genes like CO1, matK, etc. In slowly evolving organisms like plants, however, intergenic spacers (DNA regions flanked by two genes) have been under consideration as potential barcode markers, because they usually diverge faster than genes, but with conserved ends to provide primers for PCR [43], [44]. One possible factor motivating their rejection as barcode markers is their potential intractability to multiple sequence alignment, a basic step in the bioinformatics workflow within BOLD, the Barcode of Life Database. We therefore explore the potential concern that ITS might disrupt a bioinformatics workflow, by comparing the PCI derived from multiple sequence alignment to PCIs derived from other alignment methods.

## Results

### The Sequences Analyzed

This study is based on 552 *Annulohypoxylon* and *Hypoxylon* teleomorphs that we and others collected from different locations of Thailand (see Table S1 in Text S1). We classified and identified teleomorphs to species level, based on traditional morphological and anamorph characteristics, according to the holomorphic morphological species concept. For each species, we examined the relevant Thai collections, comparing to authenticated collections where necessary. Not all specimens could be cultured, not all cultures yielded successful DNA extractions, and not all extractions yielded ITS sequences under PCR, however. Large variations in morphological features and a lack of anamorph features also prevented identification of four specimens as a proper species, so they were reported as *Annulohypoxylon* spp. Phylogenetic and barcode analyses were applied to 265 ITS nrDNA sequences (see the SI), plus 4 sequences from the GenBank under *Nemania serpens*, a taxon transferred from *Hypoxylon* to the genus *Nemania* [4].

### Morphological Analysis

**Figure 1:**
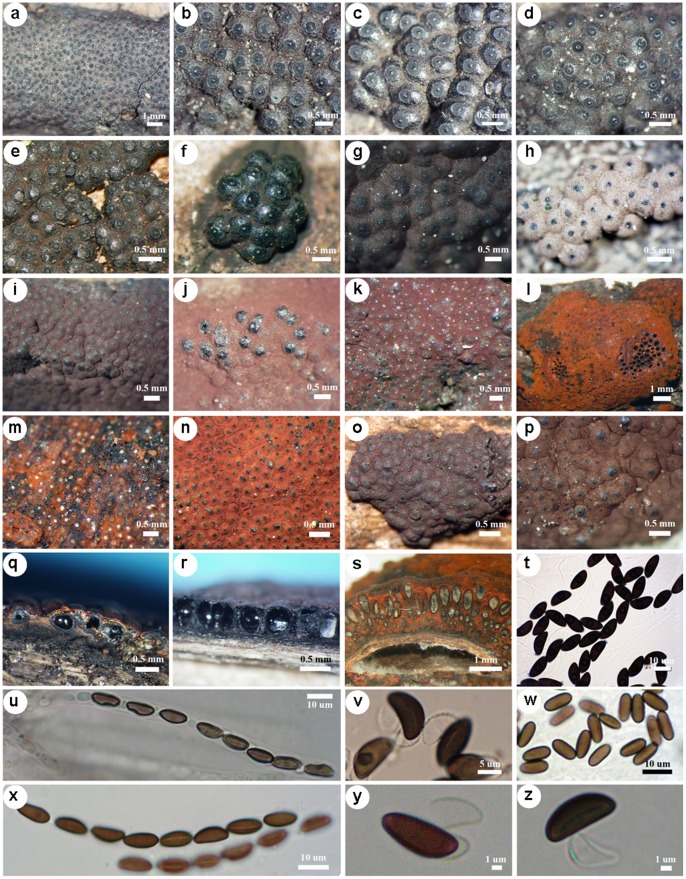
Morphological characteristics of *Annulohypoxylon* and *Hypoxylon* species found in Thailand. Stromata (a–p); perithecial structure (q–s); ascospore shapes (t–z); perispore dehiscence (v, y, z). (a) *Annulohypoxylon stygium* SUT058, (b) *A. purpureonitens* H125, (c) *A. nitens* H154, (d) *A.* aff. *nitens* H099, (e) *Annulohypoxylon* sp. H213, (f) *Annulohypoxylon* sp. H255, (g) *Hypoxylon monticulosum* H188, (h) *H*. *lenormandii* H212, (i) *H. investiens* H259, (j) *H. perforatum* SUT218, (k) *H. duranii* H250, (l) *H. haematostroma* H114, (m) *H. crocopeplum* H119, (n) *H. pelliculosum* H227, (o) *H. diatrypeoides* H226, (p) *H. rubiginosum* SUT082, (q) *H. fendleri* SUT061, (r) *H. investiens* H259, (s) *H. haematostroma* H114, (t) *H. haematostroma* SUT293, (u) *A. stygium* SUT010, (v) *H. duranii* SUT284, (w) *H. investiens* SUT041, (x) *A. nitens* SUT249, (y) *H. monticulosum* SUT185 and (z) *A. nitens* SUT025.

Figure 1 illustrates the morphological features of *Annulohypoxylon* and *Hypoxylon* species; Section “Sampling and sequencing” in the SI describes the morphometric analysis. After morphological studies, the ITS nrDNA sequences of all culturable specimens were extracted for sequence analysis.

### Sequence Analysis

The multiple alignment of ITS nrDNA had 1985 columns, reflecting many gaps in the alignment, some to accommodate a long ITS1 in occasional sequences. The alignment revealed a large variation in size ranging from 479 to 936 bp in *Annulohypoxylon* and 384 to 851 bp in *Hypoxylon*. The ITS1 fragment is extremely long (477 to 588 bp) only in the *Annulohypoxylon* species. Remarkably, the sixteen ITS sequences from *A. nitens* in our study fell into two groups. ITS1 from specimens H154, H157, H189, H197, ST2313, ST2436, ST2473 and GB15; EF026138 had lengths 159 to 163 bp, but the remaining specimens (all from Thai collections) had much longer ITS1 sequences, 543 to 572 bp. We carefully rechecked the specimens with long ITS1 sequences on teleomorph and anamorph characteristics, but they fit to *A. nitens*. Sequencing of alpha-actin and beta-tubulin [16] from both groups (H154, H157, and H189, with short ITS; H099, H181, and H215, with long ITS) supported the presence of “cryptic species” under *A. nitens*. We gave the temporary name “*A.* aff. *nitens*” to the specimens yielding a long ITS sequence, with plans to publish the new species elsewhere supported by a formal description and data from other genetic markers. (Our computer program does not accept species names containing “aff.”, so in the raw output “*A. nitensa*” replaces “*A.* aff. *nitens*”.).

### Barcode Analysis

The ITS sequences for barcode analysis consisted of two datasets. (1) The “GenBank dataset”, derived from other studies, contained 128 sequences (27 sequences from 9 *Annulohypoxylon* species, where 7 species had only one sequence; and 101 sequences from 31 *Hypoxylon* species, where 19 species had only one sequence). (2) The “non-GenBank dataset”, derived from our studies, contained 141 sequences (56 sequences from 9 *Annulohypoxylon* species, where 2 species had only one sequence; and 81 sequences from 23 *Hypoxylon* species, where 5 species had only one sequence; and 4 sequences from 1 *Nemania* species). Our “complete dataset”, which combined the GenBank and non-GenBank datasets, contained 269 sequences (83 sequences from 13 *Annulohypoxylon* species, where 5 species had only one sequence; 182 sequences from 46 *Hypoxylon* species, where 20 species had only one sequence; and 4 sequences from 1 *Nemania* species). Table S1 in Text S1 lists the samples in our complete dataset.

**Figure 2:**
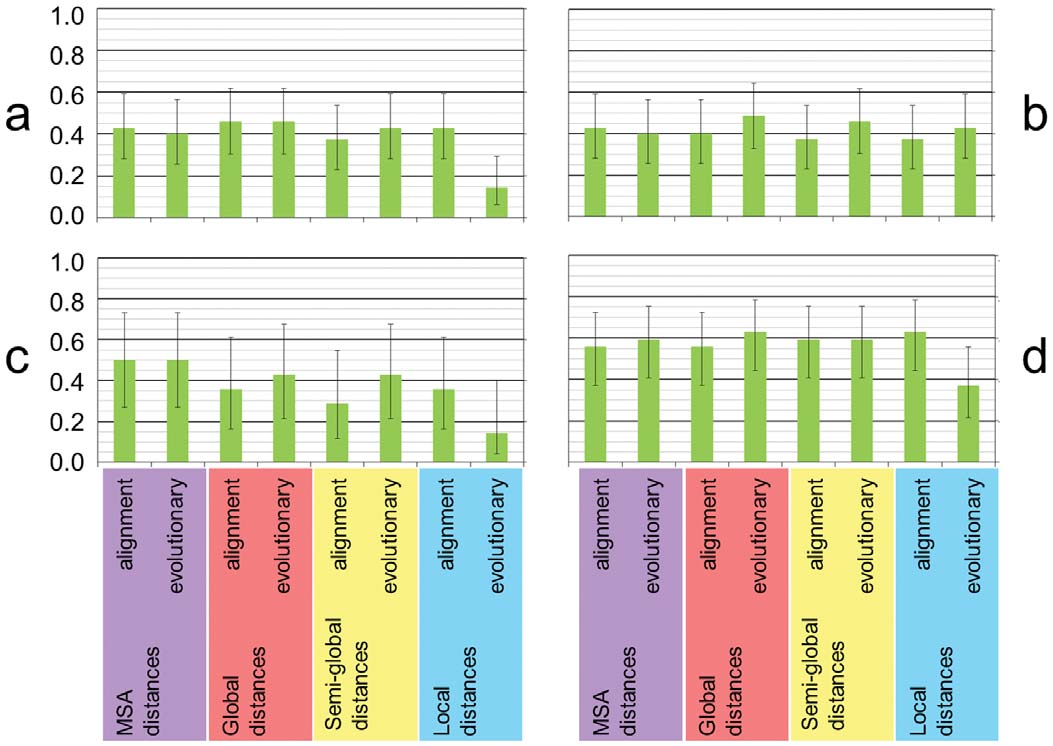
PCIs for each of four alignment types and two types of sequence distance. The error bars indicate 95% confidence intervals, as calculated by the Wilson score interval [46]. The four alignment types used (indicated by different colors at the bottom) were multiple sequence alignment (which imposes an implicit pairwise global alignment on each pair of sequences), and global, semi-global, and local pairwise alignment. The two types of sequence distance used for each alignment method were alignment distance and evolutionary distance. (In fact, for a fixed alignment type and dataset, all evolutionary distances produced the same PCI as p-distance.) The green bars give the value of the barcode gap PCI.

After the extraction of a DNA sample from any culturable specimen, the computer identified the sample’s species from its sequence using different types of alignment (e.g., local alignment; see Figure S1) and sequence distances. For each method, we calculated the barcode gap PCI [40], [41], the fraction of those species with at least two samples displaying a barcode gap [45] (see the Methods section). The Wilson score interval [46] provided the error bars in Figure 2, which display 95% confidence intervals. The Methods section describes the calculation of p-values. Figure 2 displays 32 values pertinent to correct identification of fungal species, so statistical conclusions require a multiple-test correction. The 32 values are heavily correlated, however (e.g., Figure 2c and Figure 2d represent disjoint subsets of the data; Figure 2a and Figure 2b pertain to different scoring systems aligning the same sequences; etc.). In any case, all p-values below are two-sided, stated without any multiple-testing correction, so readers can correct the p-values at their discretion (e.g., using a factor up to 32). Below, we declare a result statistically significant only at uncorrected, corresponding to the maximum number, 32, in the Bonferroni correction for multiple testing [47]. Figure 2a and Figure 2b show (barcode gap) PCIs for alignments under the NCBI BLAST [48], [49] and UCSC BLASTZ [50] default DNA scoring systems (distinguished below as “NCBI scoring” and “UCSC scoring”, respectively). Fixing the scoring system and the alignment type (either multiple sequence alignment, which imposes global pairwise alignments on each pair of sequences; or global, semi-global, or local pairwise alignment), the resulting PCIs did not depend at all on the specific evolutionary distance chosen at all, over all nine evolutionary distances examined. Under both NCBI and UCSC scoring, each PCI from global pairwise alignment never exceeded the corresponding PCI from multiple sequence alignment (MSA) by more than about 0.05, well within the sampling error of our study. The largest PCI for the entire dataset from all sequence distances was 0.37 (correct identification of 13 out of 35 species), for evolutionary distance under both global pairwise alignment and global MSA. This result is perhaps somewhat surprising, because MSAs of non-protein markers like ITS contain many gaps, degrading any evolutionary interpretation of the MSA positions. In the muscle3.6 MSA of our unique sequences, e.g., fully 208,349 of the 326,742 characters (about 64%) of the MSA consisted of gap characters.

The largest PCI differences occurred between local alignment and other alignment types, for the evolutionary distance under NCBI scoring. The PCI decrease (0.34) was statistically significant (). Semi-global alignment produced PCIs identical to global alignment, suggesting that sequence length variation had no influence on our results.

To assess the reliability of GenBank data, and to compare them to the data collected specifically for this study, we divided our dataset into “GenBank” and “non-GenBank” subsets. The best values of the PCI under both NCBI and UCSC scoring were similar, with NCBI scoring producing more varied PCIs than UCSC scoring. To examine the patterns of variation more closely, we used NCBI scoring for the remainder of the study. Thus, Figure 2c and Figure 2d compare PCIs for the GenBank and non-GenBank subsets under NCBI scoring.

The differences between the GenBank and non-GenBank sequences in number, composition, and length distribution were unremarkable (see Section “Summary statistics for the ITS sequence data” in the SI). The PCIs for the GenBank dataset are generally inferior to PCIs for non-GenBank dataset, the maximum PCI decrease being 0.56 to 0.29 for pairwise global alignment distance (not statistically significant, with ).

To assess the influence of misidentifying species morphologically, we divided our species data into two subgroups, depending on a (necessarily subjective) assessment of whether or not experts are likely to disagree on the corresponding morphological identification. We assessed likely disagreement for *Annulohypoxylon* species *annulatum*, *cohaerens*, *moriforme*, *multiforme*, *nitens*, aff. *nitens*, and *truncatum*; and for *Hypoxylon* species *anthochroum*, *crocopeplum*, *fendleri*, *macrocarpum*, *rubiginosum*, and *truncatum*. The following PCIs for multiple sequence alignment summarize the presence of a barcode gap over all species in different datasets. Table S4 in Text S1 details the PCI for the individual *Annulohypoxylon* and *Hypoxylon* species.

For the complete dataset, 13/35 species (PCI = 0.37±0.15) display a barcode gap (for all samples); when only species assessed as having consistent expert morphological identification are examined, 10/22 species (PCI = 0.45±0.19). For the GenBank dataset, 6/14 species (PCI = 0.43±0.23) display a barcode gap; when only species assessed as having consistent expert morphological identification are examined, 4/8 species (PCI = 0.50±0.29). For the non-GenBank dataset, where expert taxonomists among the authors identified the species, 15/27 species (PCI = 0.56±0.18) display a barcode gap; when only species assessed as having consistent expert morphological identification are examined, 13/20 species (PCI = 0.65±0.19). For each of the datasets (complete, GenBank, and non-GenBank), a 2×2 table was constructed, by subdividing on the likelihood of morphological misidentification by experts and on misidentification with ITS. The Fisher exact test did not indicate a significant systematic relationship between expert and ITS misidentification in any dataset (complete, 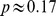; GenBank, 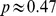; non-GenBank, 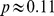) (As always, lack of significance could reflect an insufficient sample size.).

### Phylogenetic Analysis

There were 1985 positions in the multiple alignment of the ITS nrDNA sequences. The tree topology under heuristic search (with 951 informative alignment positions) did not change, whether the tree was rooted with the *N. serpens* outgroup or unrooted. More than 70% of the terminal branches received high support. Although neither *Annulohypoxylon* nor *Hypoxylon* formed a monophyletic group, many species formed highly supported monophyletic clades. Figure S2 shows the bootstrap consensus tree of 100 most parsimonious trees. It has length 7321, consistency index 0.3717, homoplasy index 0.6283 = 1–0.3717, and retention index 0.7868.

## Discussion

### Barcode Analysis

With rare but important exceptions [51], MSAs of most non-protein markers like ITS contain many gaps, and the gaps do little to elucidate biological sequence relationships. In the muscle3.6 MSA of our unique sequences, e.g., fully 64% (208,349/326,742) of the MSA characters were gap characters. Unexpectedly, however, Figure 2 indicates that even without the computational expense of calculating a phylogenetic tree, the MSA contains most of the information required to identify species. For evolutionary distances, the PCIs from an MSA are competitive with the PCIs from global pairwise alignment. When feasible alternatives exist for barcode markers in a taxon (e.g., as was the case in fungi, plants, or insects), such competitiveness can have important implications, as follows.

Presently, except for ITS, official barcode markers are exclusively protein-coding gene regions, perhaps partially reflecting a practical concern. The current bioinformatics workflow in BOLD (the Barcode of Life Database) creates MSAs with the program HMMer [52]. If for a non-protein marker, PCIs from MSAs are noticeably inferior to PCIs from other computational methods, the choice of a non- protein marker might require BOLD to redesign its bioinformatics workflow. To the contrary, however, our results suggest that even for the non- protein marker ITS, computationally rapid species identification using MSAs competes with species identification using pairwise alignment. Thus, BOLD might well be able to use non-protein barcode markers and still preserve its present bioinformatics workflow for species assignment.

Our results also reinforce the prior theoretical wisdom that global alignment is superior to local alignment in species identification [43]. On one hand, local alignment (in its so-called “linear regime” [53]) sometimes behaves like global alignment, aligning entire sequences. Indeed, under UCSC scoring, PCIs from local alignment were comparable with PCIs from global alignment. On the other hand, local alignment (in its so-called “logarithmic regime” [53]) sometimes examines only very select subsequences within a sequence pair. Under NCBI scoring, when compared to global alignment, local alignment produced a noticeably inferior PCI when based on evolutionary distance (although the PCIs based on alignment distance remained comparable).

These observations could have some relevance to barcode studies relying on the popular BLAST program for local alignment. Our results suggest that some species identifications based purely on BLAST might be more accurate if based on global alignment, particularly for non-protein markers whose alignments often contain long insertions and deletions. Note, however, that studies usually base species identifications on the BLAST similarity score (equivalent to the local alignment distance in the present context), not on evolutionary distance. The use of BLAST as a standard for comparison in bioinformatics studies assessing species identification algorithms might be suspect, however, and merits further study.

Statistically, our study could not distinguish the PCIs from global and semi-global alignment. Although computer programs implementing the Needleman-Wunsch algorithm for global alignment are readily available, our study therefore lends empirical support to substituting semi-global alignment for global alignment, if convenient.

For global alignment under NCBI and UCSC scoring, PCI differences were not significant, suggesting that at least within limits, species identification is robust against changes in the scoring system used for global sequence alignment. Our results also bear on current debate over whether the choice of evolutionary distance influences the computational efficacy of species identification with barcodes [54]–[56]. In our hands, the PCIs for all evolutionary distances were identical, indicating that the choice of evolutionary distance is immaterial to species identification with barcodes. The proportion in a pairwise alignment of nucleotide pairs consisting of different nucleotides (p-distance) is a particularly simple distance [57], so our results also support the methods of the Edinburgh conference on plant barcodes [40], which relied on p-distance without exhaustively examining alternative evolutionary distances.

In our study, non-GenBank data produced larger PCIs than GenBank data, suggesting that the GenBank entries might have contained unknown biases or even incorrect taxonomy, making them less reliable than sequences collected specifically for a barcode study. Conclusions about barcode efficacy drawn from GenBank data lacking the keyword “barcode” might therefore merit some caution.

To assess the influence of taxonomic misidentification on our analysis, we partitioned clade species on whether different experts are likely display consistency in morphological identification, i.e., on whether “experts agree” or “experts disagree”. (Like morphological identification, the partition itself is necessarily subjective.) After partitioning species by whether or not experts agree, each GenBank PCI remained less than the non-GenBank PCI. Perhaps surprisingly, the Fisher Exact test did not indicate a significant correlation between expert agreement and ITS misidentification in any of the GenBank, non-GenBank, or complete dataset (see the Results section).

Our results do suggest, however, that morphological misidentification might have lowered the absolute magnitude of PCIs for ITS. Even in the subset of species where experts agree, however, only 10/22 species were correctly identified (PCI = 0.45±0.20) in the complete dataset, and only 13/20 species (PCI = 0.65±0.19) in the non-GenBank dataset. Moreover, even under a hypothetical perfect identification of “true” species, PCIs for species where expert disagree seem unlikely to exceed PCIs for other species. Thus, the “true” PCI for the subset of non-GenBank data where experts disagree seems unlikely to exceed PCI = 0.65±0.19, and it could easily be lower, even if the Fisher Exact test was unable to detect any systematic shift.

To summarize, even the most optimistic of the PCIs we report within the clade are lower than the PCI of 0.73 reported across all fungi [36]. Our assessment of the efficacy of ITS in the *Annulohypoxylon*-*Hypoxylon* clade is therefore more pessimistic than conclusions in other studies [29]–[31]. Possible causes for our more pessimistic assessment include our relatively large sample from the clade (265 sequences for barcode analysis, compared to about 30–40 unique sequences in each of the previous studies) and the use of PCI, a stringent and numerical criterion for the success of species identification.

Within the *Annulohypoxylon*-*Hypoxylon* clade, therefore, ITS yields PCIs considered slightly low for a DNA barcode. If PCIs for *Annulohypoxylon* and *Hypoxylon* prove typical of fungal PCIs, and PCIs decrease with deeper sampling, a fungal barcode will have rely on secondary markers, just as the tentative plant barcode combining rbcL and matK did [40].

### Supplementary Phylogenetic Analyses

Based on parsimony, a phylogenetic tree recovered many *Annulohypoxylon* and *Hypoxylon* species in highly supported monophyletic clades (see Figure S2 and SI Section 3), according visually with results from the barcode PCI, and supporting the use of ITS nrDNA sequences as barcodes for these conflictive groups of fungi. The phylogenetic analysis also resolved closely related species such as *A. atroroseum* and *A. stygium*, which were separated from each other mostly by stromatal surface color and anamorph characters [5], showing that ITS aids species resolution in the *Annulohypoxylon* and *Hypoxylon* genera.

## Materials and Methods

SI Section 1 describes the collection, PCR, and sequencing of the fungal specimens. All necessary permits were obtained for the described field studies (Department of National parks, wildlife and plant conservation ref. no. 0907.1/18577).

### Sequences

The original dataset consisted of 269 FASTA records. Indeterminate identification required exclusion of four *Annulohypoxylon* sequences from the barcode analyses (see the Results section), leaving our complete dataset with 265 FASTA records and 213 unique sequences. Our analysis always retained species with only a single sample, to provide “decoys” when identifying species. Section “Summary statistics for the ITS sequence data” in the SI contains summary statistics describing the sequence data. To detect biases in sequences from GenBank, the complete dataset was also subdivided into GenBank and non-GenBank datasets.

### Assessment of Expert Morphological Identification

Expert species identification by morphology provided the gold standard in our study. To assess the magnitude of errors in our gold standard, we subdivided the species in the *Annulohypoxylon*-*Hypoxylon* clade into two groups (in the Results section), reflecting whether experts were likely to agree or disagree when identifying specimens from a species. (By necessity, the subdivision was subjective, like expert identification.) A blinded subdivision would have been ideal (with each species PCI from ITS unknown to the human judge). Because our subdivision was a response to reviewers’ comments, we did not blind it, but we recommend blinded subdivision to other investigators faced with similar issues.

### Probability of Correct Identification (PCI)

Consider any species with at least two samples. The species displays a barcode gap if its maximum intraspecific sequence distance is less than its minimum interspecific sequence distance. The barcode gap PCI is the fraction of the species (with at least two samples) that display a barcode gap [40]. Other definitions for the “correct identification” of a species are possible but less favored [41], [43].

Thus, a PCI was estimated by, the number of correctly identified species with at least two samples divided by the total number. Under a normal approximation, the estimate has a standard error mean. For our complete data set, so in the most interesting results, with, yielding.

### The Sequence Distances

#### The four alignment types

A pairwise alignment (see Figure S1) can be: (a) global, matching the whole length of two sequences [58]; (b) semi-global, matching one sequence to a subsequence of the other, and then vice versa; or (c) local, matching all subsequences of two sequences [59]. In addition, muscle3.6 aligned the sequences under its default parameters [60]. The multiple sequence alignment (MSA) contains within it an implicit but complete set of global pairwise alignments. Our study examined both NCBI and UCSC default scoring systems for DNA alignment (given in Text S1 Tables 2 and 3).

#### The two basic types of distance

For each of the four alignment types, two basic types of distance were considered. Each alignment type yielded an alignment distance (labeled “distance” in Figure 2 lumps the corresponding results together as “evolutionary”.

Thus, each of the four alignment types yielded results for the two types of distance. The same results were also computed for the GenBank and non-GenBank subsets of the complete data, using only the NCBI scoring system.

Alignment-free algorithms are simple and promise faster computation than alignment-based methods [57], [61], but presently, they have not been widely examined for species identification, so they were not explored.

### Supplementary Phylogenetic Analysis

To evaluate whether the species were recovered as monophyletic group, the multiple alignment from muscle3.6 [60] was analyzed under maximum parsimony (MP) [62] using the heuristic search option in PAUP*ver4.0b10 [63]. Two phylogenetic trees were generated, one rooted on the outgroup *Nemania serpens*, and one unrooted. Gaps were treated both as missing data and as a fifth character. Branch lengths equal to zero were collapsed to polytomies. Nonparametric bootstrap support [64] for each clade was tested with the fast-step option, using 10,000 replicates, yielding a consistency index [65], retention index [66], and homoplasy index [66]. The gaps as a fifth state degraded the phylogeny compared to gaps as missing data, so we do not present the tree corresponding to gaps as a fifth state [67].

## Supporting Information

Figure S1 A schematic diagram of three types of sequence alignments. For each of the three types of alignments diagrammed in Figure S1, the line segments represent pairs of sequences. The rectangles on the sequences represent similar pairs of subsequences. Alignments are indicated in red, with red boxes representing aligned similar subsequences; and red line segments, aligned dissimilar subsequences (which carry a penalty for the corresponding mismatches or gaps). The gray boxes represent unaligned but similar subsequences. In Figure S1a, global alignment (the Needleman-Wunsch algorithm) finds the best alignment for the entire length of the sequence pair, with penalties for gaps at the alignment ends. It therefore reflects similarity and dissimilarity throughout the full length of the sequences. In Figure S1b, semi-global alignment (a variant of the Needleman-Wunsch algorithm) finds the best alignment of the whole of second sequence against a subsequence within the first sequence, without penalizing end gaps in the other sequence. Semi-global alignment then reverses the role of the two sequences (finding the best alignment of the whole of first sequence against a subsequence within the second sequence) and returns the better of the two best alignments. In Figure S1c, local alignment (the Smith-Waterman algorithm or the heuristic BLAST algorithm) finds the best subsequence alignment within the sequence pair. Figure S1c shows that local alignment can fail to take all similarities and dissimilarities into consideration, particularly if the corresponding global alignment contains long insertions and deletions. Thus, (a) “global alignment” matches the whole length of two sequences; (b) “semi-global alignment” matches one sequence to a subsequence of the other, and then vice versa; and (c) “local alignment” matches all subsequences of two sequences.
(TIF)

Figure S2 The bootstrap consensus of the 100 most parsimonious phylogenetic trees for our *Annulohypoxylon* and *Hypoxylon* samples.
(PDF)

Text S1 Sampling and sequencing.
(PDF)

## References

[1] Tantichareon M (2004) Introduction to Thai Biodiversity. In: Jones EBG, Tanticharoen M, Hyde KD, editors. Thai Fungal Diversity BIOTECH. 1–16.

[2] Rogers JD (1994) Problem genera and family interfaces in the eupyrenomycetes. In: Hawksworth DL, editor. Ascomycete Systematics: Problems and Perspectives in the Nineties. New York, U.S.A.: Plenum Press. 321–331.

[3] Rogers JD (2000) Thoughts and musings on tropical Xylariaceae. Mycol Res 104: 1412–1420.

[4] Ju Y-M, Rogers JD (2002) The genus *Nemania* (Xylariaceae). Nova Hedwigia 74: 75–120.

[5] Ju Y-M, Rogers JD (1996) A revision of the genus *Hypoxylon* . Mycologia Mem 20: 1–365.

[6] Whalley AJS (1996) The xylariaceous way of Life. Mycological Research 100: 897–922.

[7] Whalley AJS, Phosri C, Ruchikachorn N, Sihanonth P, Sangvichien E, et al (2012) Interesting or rare Xylariaceae from Thailand. Rajabhat Journal of Science, Humanities and Social Sciences 13: 9–19.

[8] Thienhirun S, Whalley AJS (2004) Xylariaceae. In: Jones EBG, Tanticharoen M, Hyde KD, editors. Thai Fungal Diversity: BIOTECH. 71–77.

[9] Lumbsch T, Hundorf S (2010) Outline of the Ascomycota (2009). Life and Earth Sciences: Fieldiana. 1–64.

[10] Fournier J, Stadler M, Hyde KD, Duong ML (2010) The new genus *Rostrohypoxylon* and two new *Annulohypoxylon* species from Northern Thailand Fungal Divers. 40: 23–36.

[11] Suwannasai N, Rodtong S, Thienhirun S, Whalley AJS (2005) New species and phylogenetic relationships of *Hypoxylon* species found in Thailand inferred from the internal transcribed spacer regions of ribosomal DNA sequences. Mycotaxon 94: 303–324.

[12] Læssøe T, Srikitikulchai P, Fournier J, Kopcke B, Stadler M (2010) Lepraric acid derivatives as chemotaxonomic markers in *Hypoxylon aeruginosum*, *Chlorostroma subcubisporum* and *C. cyaninum*, sp. nov. Fungal Biol 114: 481–489. 20943159 10.1016/j.funbio.2010.03.010

[13] Læssøe T, Rogers JD, Whalley AJS (1989) *Camillea*, *Jongiella* and light-spored species of *Hypoxylon* . Mycol Res 93: 121–155.

[14] Rogers JD (1977) Surface features of light-colored ascospores of some applanate *Hypoxylon* species. Canad J Bot 55: 2394–2398.

[15] Whalley MA (1996) Distinctive features of *Camillea* (Xylariaceae) from Cuyabeno as revealed by scanning electron microscopy. The Mycologist 10: 149–151.

[16] Hsieh HM, Ju YM, Rogers JD (2005) Molecular phylogeny of *Hypoxylon* and closely related genera. Mycologia 97: 844–865. 16457354 10.3852/mycologia.97.4.844

[17] Pelaez F, Gonzalez V, Platas G, Sanchez-Ballesteros J, Rubio V (2008) Molecular phylogenetic studies within the Xylariaceae based on ribosomal DNA sequences. Fungal Divers 31: 111–134.

[18] Stadler M, Fournier J (2006) Pigment chemistry, taxonomy and phylogeny of the Hypoxyloideae (Xylariaceae). Rev Iberoam Micol 23: 160–170. 17196023 10.1016/s1130-1406(06)70037-7

[19] Stadler M, Hellwig V (2005) Chemotaxonomy of the Xylariaceae and remarkable bioactive compounds from *Xylariales* and their associated asexual stages. Recent Res Develop Phytochem 9: 41–93.

[20] Tang AMC, Jeewon R, Hyde KD (2009) A re-evaluation of the evolutionary relationships within the Xylariaceae based on ribosomal and protein-coding gene sequences. Fungal Divers 34: 155–153.

[21] Taylor JW, Jacobson DJ, Kroken S, Kasuga T, Geiser DM, et al (2000) Phylogenetic species recognition and species concepts in fungi. Fungal Genetics and Biology 31: 21–32. 11118132 10.1006/fgbi.2000.1228

[22] Okane I, Srikitikulchai P, Toyama K, Læssøe T, Sivichai S, et al (2008) Study of endophytic Xylariaceae in Thailand: diversity and taxonomy inferred from rDNA sequence analyses with saprobes forming fruit bodies in the field. Mycoscience 49: 359–372.

[23] Pinnoi A, Phongpaichit P, Jeewon R, Tang AMC, Hyde KD, et al (2010) Phylogenetic relationships of *Astrocystis eleiodoxae* sp. nov. (Xylariaceae). Mycosphere 1: 1–9.

[24] Thienhirun S (1997) A Preliminary account of the Xylariaceae of Thailand. Liverpool Liverpool John Moores University.

[25] Hebert PD, Cywinska A, Ball SL, deWaard JR (2003) Biological identifications through DNA barcodes. Proc Biol Sci 270: 313–321. 12614582 10.1098/rspb.2002.2218 PMC1691236

[26] Floyd R, Abebe E, Papert A, Blaxter M (2002) Molecular barcodes for soil nematode identification. Mol Ecol 11: 839–850. 11972769 10.1046/j.1365-294x.2002.01485.x

[27] Brock PM, Doring H, Bidartondo MI (2009) How to know unknown fungi: the role of a herbarium. New Phytol 181: 719–724. 19076294 10.1111/j.1469-8137.2008.02703.x

[28] Gardes M, Fortin J, White TJ, Bruns TD, Taylor JW (1991) Identification of indigenous and introduced symbiotic fungi in ectomycorrhizae by amplification of nuclear and mitochondrial ribosomal DNA. Canad J Bot 69: 180–190.

[29] Sanchez-Ballesteros J, Gonzalez V, Salazar O, Acero J, Portal MA, et al (2000) Phylogenetic study of *Hypoxylon* and related genera based on ribosomal ITS sequences. Mycologia 92: 964–977.

[30] Triebel D, Persoh D, Wollweber H, Stadler M (2005) Phylogenetic relationships among *Daldinia*, *Entonaema*, and *Hypoxylon* as inferred from ITS nrDNA analyses of Xylariales. Nova Hedwigia 80: 25–43.

[31] Lee JS, Ko KS, Jung HS (2000) Phylogenetic analysis of *Xylaria* based on nuclear ribosomal ITS1–5. 8S-ITS2 sequences. FEMS Microbiol Lett 187: 89–93. 10828406 10.1111/j.1574-6968.2000.tb09142.x

[32] Begerow D, Nilsson H, Unterseher M, Maier W (2010) Current state and perspectives of fungal DNA barcoding and rapid identification procedures. Appl Microbiol Biotechnol 87: 99–108. 20405123 10.1007/s00253-010-2585-4

[33] El Karkouri K, Murat C, Zampieri E, Bonfante P (2007) Identification of internal transcribed spacer sequence motifs in truffles: a first step toward their DNA bar coding. Appl Environ Microbiol 73: 5320–5330. 17601808 10.1128/AEM.00530-07 PMC1950968

[34] Feau N, Vialle A, Allaire M, Tanguay P, Joly DL, et al (2009) Fungal pathogen (mis-)identifications: a case study with DNA barcodes on *Melampsora* rusts of aspen and white poplar. Mycol Res 113: 713–724. 19249365 10.1016/j.mycres.2009.02.007

[35] Nguyen HD, Seifert KA (2008) Description and DNA barcoding of three new species of Leohumicola from South Africa and the United States. Persoonia 21: 57–69. 20396577 10.3767/003158508X361334 PMC2846127

[36] Schoch CL, Seifert KA, Huhndorf S, Robert V, Spouge JL, et al (2012) Nuclear ribosomal internal transcribed spacer (ITS) region as a universal DNA barcode marker for Fungi. Proc Natl Acad Sci U S A 109: 6241–6246. 22454494 10.1073/pnas.1117018109 PMC3341068

[37] Chase MW, Salamin N, Wilkinson M, Dunwell JM, Kesanakurthi RP, et al (2005) Land plants and DNA barcodes: short-term and long-term goals. Philos Trans R Soc Lond B Biol Sci 360: 1889–1895. 16214746 10.1098/rstb.2005.1720 PMC1609218

[38] Cowan RS, Chase MW, Kress JW, Savolainen V (2006) 300,000 species to identify: problems, progress, and prospects in DNA barcoding of land plants. Taxon 55: 611–616.

[39] Kress WJ, Erickson DL (2008) DNA barcodes: genes, genomics, and bioinformatics. Proc Natl Acad Sci U S A 105: 2761–2762. 18287050 10.1073/pnas.0800476105 PMC2268532

[40] CBOL Plant Working Group (2009) A DNA barcode for land plants. Proc Natl Acad Sci U S A 106: 12794–12797. 19666622 10.1073/pnas.0905845106 PMC2722355

[41] Meier R, Shiyang K, Vaidya G, Ng PK (2006) DNA barcoding and taxonomy in Diptera: a tale of high intraspecific variability and low identification success. Syst Biol 55: 715–728. 17060194 10.1080/10635150600969864

[42] Huang D, Meier R, Todd PA, Chou LM (2008) Slow mitochondrial COI sequence evolution at the base of the metazoan tree and its implications for DNA barcoding. J Mol Evol 66: 167–174. 18259800 10.1007/s00239-008-9069-5

[43] Erickson DL, Spouge JL, Resch A, Weight LA, Kress JW (2008) DNA barcoding in land plants: developing standards to quantify and maximize success. Taxon 13: 1304–1316. PMC2749701 19779570

[44] Kress WJ, Erickson DL (2007) A Two-Locus Global DNA Barcode for Land Plants: The Coding rbcL Gene Complements the Non-Coding trnH-psbA Spacer Region. PLoS ONE 2: e508. 17551588 10.1371/journal.pone.0000508 PMC1876818

[45] Hebert PD, Stoeckle MY, Zemlak TS, Francis CM (2004) Identification of Birds through DNA Barcodes. PLoS Biol 2: e312. 15455034 10.1371/journal.pbio.0020312 PMC518999

[46] Wilson EB (1927) Probable inference, the law of succession, and statistical inference. Journal of the American Statistical Association 22: 209–212.

[47] Holm S (1979) A simple sequentially rejective multiple test procedure. Scand J Statist 6: 65–70.

[48] Altschul SF, Madden TL, Schaffer AA, Zhang J, Zhang Z, et al (1997) Gapped BLAST and PSI-BLAST: a new generation of protein database search programs. Nucleic Acids Res 25: 3389–3402. 9254694 10.1093/nar/25.17.3389 PMC146917

[49] Altschul S (1999) Hot papers - Bioinformatics - Gapped BLAST and PSI-BLAST: a new generation of protein database search programs by S.F. Altschul, T.L. Madden, A.A. Schaffer, J.H. Zhang, Z. Zhang, W. Miller, D.J. Lipman - Comments. Scientist 13: 15–15. 10.1093/nar/25.17.3389 PMC146917 9254694

[50] Schwartz S, Kent WJ, Smit A, Zhang Z, Baertsch R, et al (2003) Human-mouse alignments with BLASTZ. Genome Research 13: 103–107. 12529312 10.1101/gr.809403 PMC430961

[51] Bejerano G, Pheasant M, Makunin I, Stephen S, Kent WJ, et al (2004) Ultraconserved elements in the human genome. Science 304: 1321–1325. 15131266 10.1126/science.1098119

[52] Eddy SR (1995) Multiple alignment using hidden Markov models. Proc Int Conf Intell Syst Mol Biol 3: 114–120. 7584426

[53] Arratia R, Waterman MS (1985) Critical Phenomena in Sequence Matching. Annals of Probability 13: 1236–1249.

[54] Kwong S, Srivathsan A, Vaidya G, Meier R (2012) Is the COI barcoding gene involved in speciation through intergenomic conflict? Mol Phylogenet Evol 62: 1009–1012. 22182989 10.1016/j.ympev.2011.11.034

[55] Fregin S, Haase M, Olsson U, Alstrom P (2012) Pitfalls in comparisons of genetic distances: a case study of the avian family Acrocephalidae. Mol Phylogenet Evol 62: 319–328. 22023826 10.1016/j.ympev.2011.10.003

[56] Collins RA (2012) Barcoding’s next top model: an evaluation of nucleotide substitution models for specimen identification. Methods in Ecology and Evolution.

[57] Little DP, Stevenson DW (2007) A comparison of algorithms for the identification of specimens using DNA barcodes: examples from gymnosperms. Cladistics 23: 1–27. 10.1111/j.1096-0031.2006.00126.x 34905841

[58] Needleman SB, Wunsch CD (1970) A general method applicable to the search for similarities in the amino acid sequence of two proteins. J Mol Biol 48: 443–453. 5420325 10.1016/0022-2836(70)90057-4

[59] Smith TF, Waterman MS (1981) Identification of common molecular subsequences. J Mol Biol 147: 195–197. 7265238 10.1016/0022-2836(81)90087-5

[60] Edgar RC (2004) MUSCLE: a multiple sequence alignment method with reduced time and space complexity. BMC Bioinformatics 5: 113. 15318951 10.1186/1471-2105-5-113 PMC517706

[61] Kuksa P, Pavlovic V (2009) Efficient alignment-free DNA barcode analytics. BMC Bioinformatics 10: Article No.: S9. 10.1186/1471-2105-10-S14-S9 PMC2775155 19900305

[62] Larget B, Simon DL (1999) Markov chain Monte Carlo algorithms for the Bayesian analysis of phylogenetic trees. Mol Biol Evol 16: 750–759.

[63] Swofford DL (2003) PAUP*, Phylogenetic analysis using parsimony (*and other methods). Sunderland, Massachusetts: Sinauer Association Inc. 303–324 p.

[64] Felsenstein J (1985) Confidence limits on phylogenies: an approach using the bootstrap. Evolution 39: 783–791. 28561359 10.1111/j.1558-5646.1985.tb00420.x

[65] Kluge AG, Farris JS (1969) Quantitative phyletics and the evolution of anurans. Syst Zool 18: 1–32.

[66] Farris JS (1989) The retention index and the rescaled consistency index. Cladistics 5: 417–419. 10.1111/j.1096-0031.1989.tb00573.x 34933481

[67] Ogden TH, Rosenberg MS (2007) How should gaps be treated in parsimony? A comparison of approaches using simulation. Mol Phylogenet Evol 42: 817–826. 17011794 10.1016/j.ympev.2006.07.021

