# Supplementary figures and images for: Fungi in Thailand: A Case Study of the Efficacy of an ITS Barcode for Automatically Identifying Species within the Annulohypoxylon and Hypoxylon Genera
Source: PLoS One. 2013 Feb 4;8(2):e54529. doi: 10.1371/journal.pone.0054529 (PMC3563529; doi:10.1371/journal.pone.0054529)

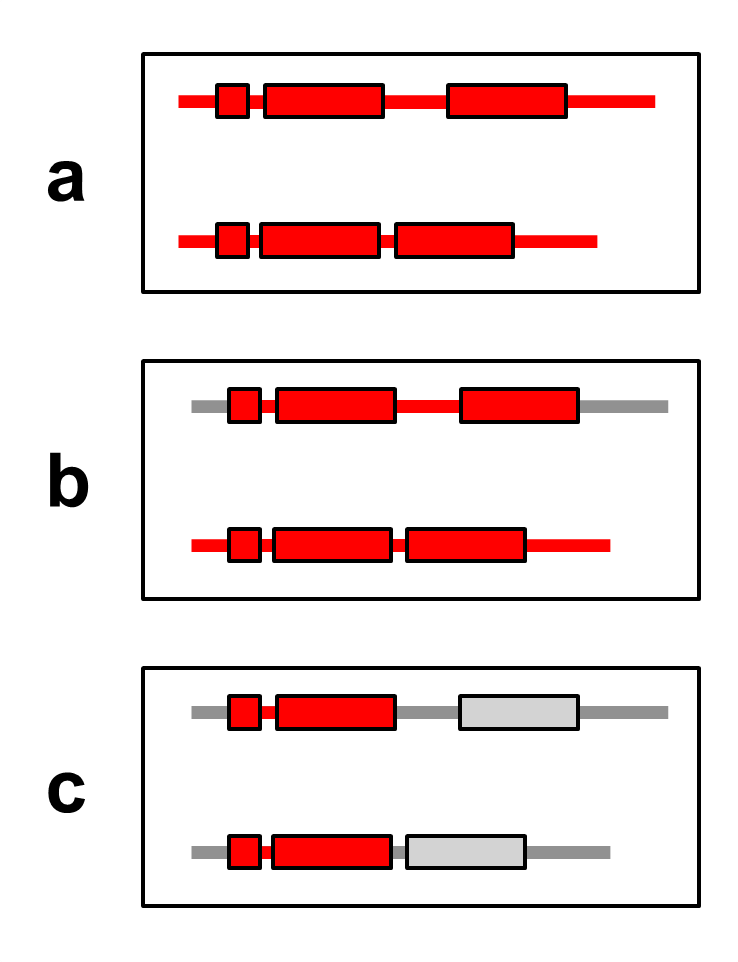

Supplement: Figure S1 — A schematic diagram of three types of sequence alignments. For each of the three types of alignments diagrammed in Figure S1, the line segments represent pairs of sequences. The rectangles on the sequences represent similar pairs of subsequences. Alignments are indicated in red, with red boxes representing aligned similar subsequences; and red line segments, aligned dissimilar subsequences (which carry a penalty for the corresponding mismatches or gaps). The gray boxes represent unaligned but similar subsequences. In Figure S1a, global alignment (the Needleman-Wunsch algorithm) finds the best alignment for the entire length of the sequence pair, with penalties for gaps at the alignment ends. It therefore reflects similarity and dissimilarity throughout the full length of the sequences. In Figure S1b, semi-global alignment (a variant of the Needleman-Wunsch algorithm) finds the best alignment of the whole of second sequence against a subsequence within the first sequence, without penalizing end gaps in the other sequence. Semi-global alignment then reverses the role of the two sequences (finding the best alignment of the whole of first sequence against a subsequence within the second sequence) and returns the better of the two best alignments. In Figure S1c, local alignment (the Smith-Waterman algorithm or the heuristic BLAST algorithm) finds the best subsequence alignment within the sequence pair. Figure S1c shows that local alignment can fail to take all similarities and dissimilarities into consideration, particularly if the corresponding global alignment contains long insertions and deletions. Thus, (a) “global alignment” matches the whole length of two sequences; (b) “semi-global alignment” matches one sequence to a subsequence of the other, and then vice versa; and (c) “local alignment” matches all subsequences of two sequences. (TIF) [file pone.0054529.s001.tif]

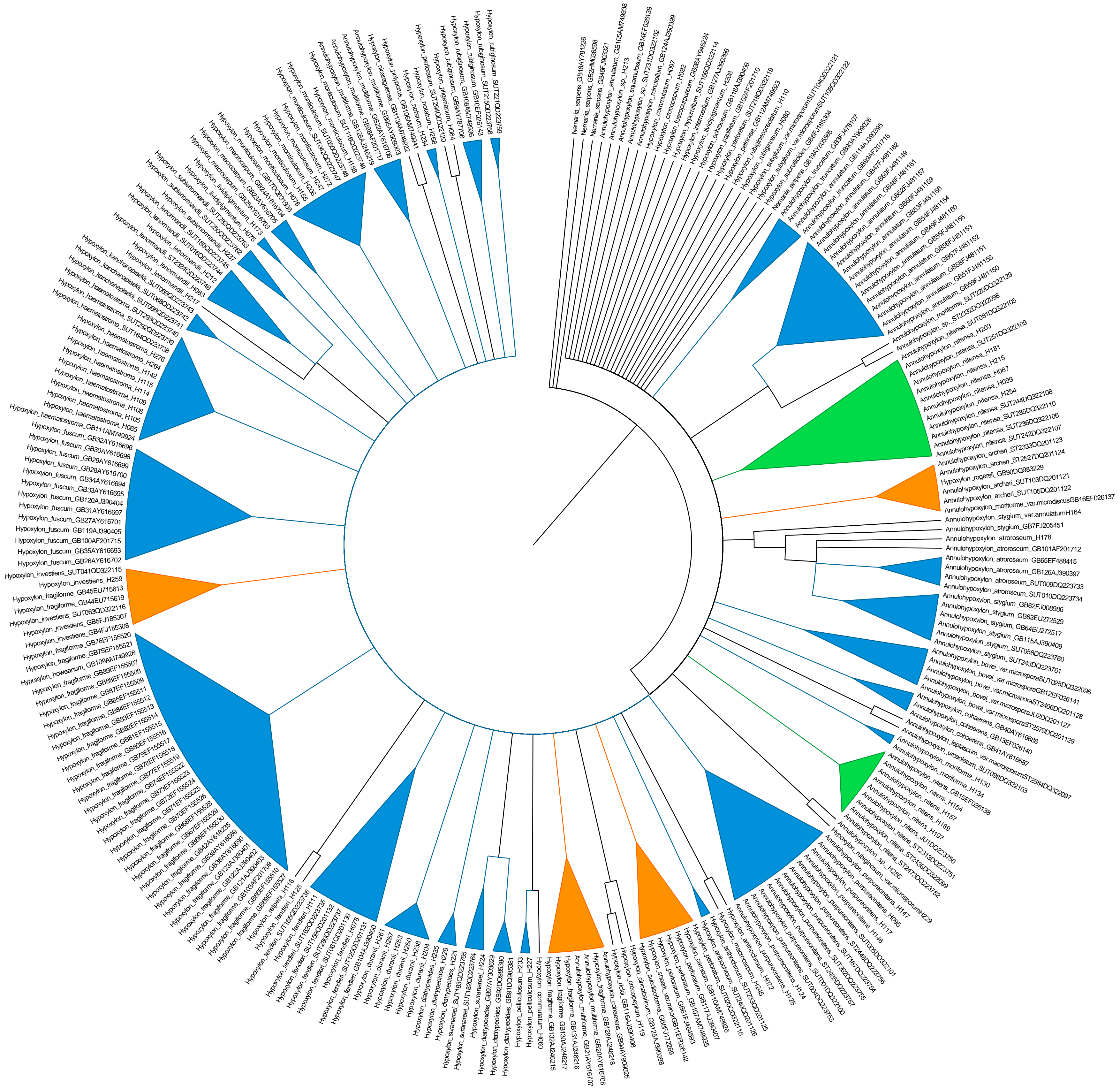

Supplement: Figure S2 — The bootstrap consensus of the 100 most parsimonious phylogenetic trees for our Annulohypoxylon and Hypoxylon samples. (PDF) [file pone.0054529.s002.pdf]
