# Supplementary material for: Fungi in Thailand: A Case Study of the Efficacy of an ITS Barcode for Automatically Identifying Species within the Annulohypoxylon and Hypoxylon Genera
Source: PLoS One. 2013 Feb 4;8(2):e54529. doi: 10.1371/journal.pone.0054529 (PMC3563529; doi:10.1371/journal.pone.0054529)
Supplement: Text S1 — Sampling and sequencing. (PDF) [file pone.0054529.s003.pdf]

Sampling and sequencing

Sampling and morphometric analysis

Fungal specimens were collected throughout Thailand. The date and location of all collections, including type of host, were recorded. Fresh stromata were photographed and described, and if appropriate, spores were removed and transferred onto Potato Dextrose Agar (PDA) for culturing. The morphological characteristics of the spores such as shape, size, and color of stromata, perithecia, ascospores, etc. were observed [1-4]. The color reaction between 10% Potassium hydroxide (KOH) and stromatal surface was performed and compared to the mycological color chart [5]. Ascospores mounted in Melzer's reagent were examined for characteristics such as the type of germ slit and then photographed by light microscopy (Olympus BX-40). Perispores and epispores in 10% KOH were observed. The size range of the spores was calculated from measurements of the width and length of at least thirty spores. Any ornamentation present was recorded and later critically analyzed using scanning electron microscopy (SEM, JEOL-840). All fungal collections are located at the Microbiology Programme Herbarium, Faculty of Science and Technology, Pibulsongkram Rajabhat University, Phitsanulok, Thailand.

PCR and sequencing

Fungal mycelium was harvested from pure cultures on PDA. Mycelium was washed twice with sterile distilled water, frozen at -70°C and ground with a micro pestle. Fungal genomic DNA was extracted following the instructions of a DNeasy Plant Mini Kit (QIAGEN, Valencia, California, USA). The ITS nrDNA was amplified by using ITS5/ITS4 as forward and reverse primers, respectively (66). PCR reactions were carried out in a total volume of 50 μl containing 100 ng DNA template, 1 X PCR buffer and 1.5 mM $MgCl_2$, 2 mM dNTPs, 0.2 μM of each primer and 0.5 units of Taq Polymerase (Invitrogen, USA). Thermal cycling parameters were as following; initial denaturation at 95 ^{o}C for 5 mins, followed by 30 cycles of 95 ^{o}C for 30 sec, 55 ^{o}C for 30 sec, 72 ^{o}C for 1 min and a final elongation for 7-10 mins at 72 ^{o}C . Further problematic isolates were also studied on the partial region of α-actin and β-tubulin genes using ACT512F/ACT783R (67) and T1/T22 [6] as primers, respectively. The parameters of thermal cycling were as following; initial denaturation at 95 ^{o}C for 5 mins, followed by 30 cycles of 95 ^{o}C for 1 min, 50 ^{o}C for 1.5 min, 72 ^{o}C for 2 min and a final elongation for 10 mins at 72 ^{o}C. The PCR products were checked in 1 % agarose gel electrophoresis before purified with QIAquick PCR Purification Kit (QIAGEN). They were then directly sequenced with BigDye on an ABI 377 automated sequencer (Perkin Elmer, USA) using the same primers as for amplification. Each sequence was manually checked for ambiguous bases and the two sequences (direct and reverse) were assembled with Bioedit 5.0.6 (69).

Summary statistics for the ITS sequence data

The original dataset consisted of 269 FASTA records. As described at the start of the Results section, indeterminate identification required exclusion of four *Annulohypoxylon* sequences from

the barcode analyses, leaving our complete dataset with 265 FASTA records and 213 unique sequences.

Within the complete dataset, there were 94 samples from 16 of the 38 *Annulohypoxylon* species, 167 samples from 41 of the 112 *Hypoxylon* species, and 4 sequences under *Nemania serpens*. Of the 16 *Annulohypoxylon* species, 12 had at least 2 samples; of the 41 *Hypoxylon* species, 23. Sequence lengths ranged from 384 to 936, with the mean ± standard error equaling 556±116.

To detect biases in sequences from GenBank, the complete dataset was subdivided into GenBank and non-GenBank datasets.

The GenBank dataset consisted of 128 FASTA records with 103 unique sequences. There were 42 samples from 11 *Annulohypoxylon* species, 82 samples from 25 *Hypoxylon* species and the 4 *Nemania serpens* samples. Of the 11 *Annulohypoxylon* species, 6 had at least 2 samples; of the *Hypoxylon* species, 8 had at least 2 samples. Sequence lengths ranged from 384 to 936, with the mean ± standard error equaling 547±96.

The non-GenBank dataset contained 137 FASTA records with 110 unique sequences. There were 52 samples from 10 *Annulohypoxylon* species and 85 samples from 24 *Hypoxylon* species. Of the 10 *Annulohypoxylon* species, 8 had at least 2 samples; of the *Hypoxylon* species, 19 had at least 2 samples. Sequence lengths ranged from 444 to 916, with the mean ± standard error equaling 567±132.

In both GenBank and non-GenBank datasets, each unambiguous nucleotide (A,C,G,T) constituted an unremarkable 22% to 28% of the unique sequences; all ambiguous nucleotides together, less than 0.1%.

Supplementary phylogenetic analyses

The phylogenetic tree recovered many *Annulohypoxylon* and *Hypoxylon* species in highly supported monophyletic clades, supporting the use of ITS nrDNA sequences as barcodes for these conflictive groups of fungi. *N. serpens* proved best of several outgroups tried for the tree, although the tree placement of the *Annulohypoxylon* and *Hypoxylon* genera included the *N. serpens* sample AY805565 (from GenBank, and therefore lacking a standard voucher). Most sequences in the blue clade contained only one "species name" such as *A. annulatum*, *A. atroroseum*, *A. bovei* var. *microspora*, *A. cohaerens*, *A. multiforme*, *A. nitens*, *A. purpureonitens*, *A. stygium*, *A. truncatum*, *H. diatrypeoides*, *H. duranii*, *H. fendleri*, *H. fragiforme*, *H. fuscum*, *H. haematrostroma*, *H. investiens*, *H. lenormandii*, *H. macrocarpum*, *H. monticulosum*, and *H. sublenormandii*. Fourteen sequences of *A. annulatum* were grouped together and shared the same host of *Tremella fuciformis* noted from China except that two sequences of *A. annulatum* (GB114; AJ390395 and GB105; AM749938) were segregated in another two different clades. One *A. annulatum* sequence (GB105; AM749938) exhibited the extremely long ITS1 region (584 bp) which is similar to those found in *A. atroroseum*, *A. stygium* and "*A.* aff. *nitens*" specimens, whereas another *A. annulatum* sequence (GB114; AJ390395) showed the common size (179 bp). There were three *Annulohypoxylon truncatum* sequences grouped together

including one *A. annulatum* sequence (GB114; AJ390395), which had identical sequences to *A. truncatum* of up to 99% similarity. In the *Annulohypoxylon cohaerens* clade one sequence of *A. minutellum* (GB124: AJ390399) was included with three sequences of *A. cohaerens*. This result indicates the close relationship within this group since *A. minutellum* has been published as a synonym of *A. cohaerens* var. *microsporum* [7]. It has 87% similarity to all *A. cohaerens* sequences.

This phylogenetic analysis also resolved closely related species such as *A. atroroseum* and *A. stygium*, which are basically separated from each other by stromatal surface color and anamorph characters [2]. They were clearly separated by ITS nrDNA sequences. Similarly, *A. bovei* var. *microspora*, *A. moriforme* and *A. nitens* are quite close in morphological characters and presence of a *Nodulisporium*-like anamorph, but their ITS sequences distinguished them. The phylogenetic tree in Figure S2 also clearly separates the "cryptic species" *A. nitens* and "*A.* aff. *nitens*" (green clades). Under global alignment within an MSA, each of *A. nitens* and "*A.* aff. *nitens*" was correctly identified monophyletically, so as a barcode marker, ITS separates the cryptic species found in this study. Some species that possess distinct and generally recognized characters such as *A. purpureonitens*, *H. haematrostroma*, *H. lenormandii*, *H. sublenormandii* and *H. monticulosum* exhibited clear species separation in each of the clades. Particularly, *H. fragiforme* the type species of the genus *Hypoxylon* was commonly studied and had the highest numbers of ITS nrDNA sequences available in GenBank as shown in a large blue clade (29 sequences). However, another eight sequences of *H. fragiforme* were placed in 3 different clades. Four sequences (GB129; AJ246218, GB130; AJ246217, GB131; AJ246216 and GB132; AJ246215) grouped in a complex clade, which contained another 4 different species. Two *H. fragiforme* sequences (GB44; EU715619 and GB45; EU715613) were included in the *H. investiens* group, whereas the last two *H. fragiforme* sequences (GB86; EF155510 and GB69; EF1555527) were individually separated. In the case of *H. fendleri,* although the specimens studied revealed high variation in minor morphological characters as described above, they still grouped together in both the GenBank and non-GenBank datasets.

In the *A. archeri* clade most members belonged to the non-GenBank dataset, except for one sequences of *A. moriforme* var. *microdiscus* (GB16; EF026137). Both species are very close in morphological characters except that perithecial diameter of *A. archeri* (0.1-0.3 mm) is smaller than that of *A. moriforme* var. *microdiscus* (> 0.3 mm) [2]. They do however lack anamorph character information. Also a recent study of *alpha*-actin and *beta*-tubulin genes within *Hypoxylon* and closely related genera recognized only *A. moriforme* var. *microdiscus* [7]. Both species need more collections and extensive study of both teleomorph and anamorph characters to confirm or reject this conclusion.

Several clades of sequences containing species complexes are placed in orange clades. The largest of these groups included the five members of *H. perforatum* (GB107; AM749935, GB61; FJ464593, GB117; AJ390407, QD322118 and QD322120), *H. carneum* (GB110; AM749926), *H. shearii* var. *minor* (GB11; EF026142), *H. subdisciforme* (GB8; FJ172269: unpublished species) and *H.* cf. *pilgerianum* (H064). Although the *H. perforatum* sequences from both GenBank and non-GenBank datasets placed in this clade it also included four other species. The sequence of *H. carneum* (GB110; AM749926) was 99% similar to *H. perforatum* (GB107; AM749935), and both of them had the same chemotype (63). However, ITS sequences did not

support the species identification within this complex clade: *H. fragiforme* (GB129; AJ246218, GB130; AJ246217, GB131; AJ246216 and GB132; AJ246215), *A. cohaerens* (GB94; AY909025) and *A. multiforme* (GB20; AY616708 and GB21; AY616707) had similar sequences, exhibiting 98% sequence identity. In contrast, in another complex clade, *H. crocopeplum* (H119), *H. rickii* (GB116; AJ390408) and *H. cinnabarinum* (GB125; AJ390398) were different in many characters, and their ITS sequences showed up to 12% divergence. In yet another complex clade, consisting of *H. rubiginosum* (H080) and *H. subgilvum* var. *microsporum* (SUT108QD322122), there were two different sequences from two different specimens, indicating a need to examine more specimens.

Table S1: Fungal taxa used in this study and their accession numbers **(**"*A.* aff. *nitens*" as a provisional name)

Taxon	Code	Accession No.		Fungal information
		GenBank	Our study	
Annulohypoxylon annulatum	GB47	FJ481162	-	Fuyang Town, Bazhong City, Sichuan Province, China
A. annulatum	GB48	FJ481161	-	Fuyang Town, Bazhong City, Sichuan Province, China
A. annulatum	GB49	FJ481160	-	Fuyang Town, Bazhong City, Sichuan Province, China
A. annulatum	GB50	FJ481159	-	Fuyang Town, Bazhong City, Sichuan Province, China
A. annulatum	GB51	FJ481158	-	Yangbo Town, Bazhong City, Sichuan Province, China
A. annulatum	GB52	FJ481157	-	Nuoshuihe Town, Bazhong City, Sichuan Province, China
A. annulatum	GB53	FJ481156	-	Nuoshuihe Town, Bazhong City, Sichuan Province, China
A. annulatum	GB54	FJ481154	-	Shizi Mountain of Chengdu City, Sichuan Province, China
A. annulatum	GB55	FJ481155	-	Nuoshuihe Town, Bazhong City, Sichuan Province, China
A. annulatum	GB56	FJ481153	-	Shizi Mountain of Chengdu City, Sichuan Province, China
A. annulatum	GB57	FJ481152	-	Shizi Mountain of Chengdu City, Sichuan Province, China
A. annulatum	GB58	FJ481151	-	Shizi Mountain of Chengdu City, Sichuan Province, China
A. annulatum	GB59	FJ481150	-	Shizi Mountain of Chengdu City, Sichuan Province, China
A. annulatum	GB60	FJ481149	-	Shizi Mountain of Chengdu City, Sichuan Province, China
A. annulatum	GB105	AM749938	-	-
A. annulatum	GB114	AJ390395	-	USA
A. archeri	SUT103	-	DQ201121	Songkhla Province, Thailand
A. archeri	SUT105	-	DQ201122	Songkhla Province, Thailand
A. archeri	ST2333	-	DQ201123	Royal Forest Department, Bangkok, Thailand
A. archeri	ST2527	-	DQ201124	Royal Forest Department, Bangkok, Thailand
A. atroroseum	GB65	EF488415	-	China
A. atroroseum	GB101	AF201712	-	-
A. atroroseum	H178	-	FN252415	Phitsanulok Province, Thailand
A. atroroseum	SUT009	-	DQ223733	Nakhon Ratchasima Province, Thailand
A. atroroseum	SUT010	-	DQ223734	Nakhon Ratchasima Province, Thailand
A. atroroseum	GB126	AJ390397	-	USA

A. bovei var. *microspora*	GB12	EF026141	-	Taiwan
A. bovei var. *microspora*	SUT025	-	DQ322096	Chaiyaphum Province, Thailand
A. bovei var. *microspora*	ST2579	-	DQ201129	Royal Forest Department, Bangkok, Thailand
A. bovei var. *microspora*	ST2406	-	DQ201128	Royal Forest Department, Bangkok, Thailand
A. bovei var. *microspora*	JU2	-	DQ201127	Taiwan
A. cohaerens	GB13	EF026140	-	France
A. cohaerens	GB40	AY616688	-	-
A. cohaerens	GB41	AY616687	-	-
A. cohaerens	GB94	AY909025	-	-
A. leptascum var. *macrosporum*	ST2584	-	DQ322097	Royal Forest Department, Bangkok, Thailand
A. minutellum	GB124	AJ390399	-	USA
A. moriforme	SUT220	-	DQ322129	Trad Province, Thailand
A. moriforme	H130	-	FN252424	Phitsanulok Province, Thailand
A. moriforme	H134	-	FN252425	Phitsanulok Province, Thailand
A. moriforme var. *microdiscus*	GB16	EF026137	-	Isolate 90080807; Taiwan
A. multiforme	GB20	AY616708	-	-
A. multiforme	GB21	AY616707	-	-
A. multiforme	GB22	AY616706	-	-
A. multiforme	GB95	AY909003	-	-
A. multiforme	GB98	AF201717	-	-
A. multiforme	GB128	AJ246219	-	United Kingdom
A. nitens	GB15	EF026138	-	Isolate 91022108; Taiwan
A. nitens	H154	-	FM209453	Chiang Rai Province, Thailand
A. nitens	H157	-	FM209455	Chiang Rai Province, Thailand
A. nitens	H189	-	FM209459	Phitsanulok Province, Thailand
A. nitens	H197	-	FM209461	Phitsanulok Province, Thailand
A. nitens	ST2313	-	DQ223751	Royal Forest Department, Bangkok, Thailand
A. nitens	ST2436	-	DQ322099	Royal Forest Department, Bangkok, Thailand
A. nitens	ST2473	-	DQ223752	Royal Forest Department, Bangkok, Thailand
A. nitens	JU1	-	DQ223750	Taiwan
A. aff. *nitens*	H087	-	FN252418	Phitsanulok Province, Thailand
A. aff. *nitens*	H099	-	FN252419	Phitsanulok Province, Thailand
A. aff. *nitens*	H181	-	FN252420	Phitsanulok Province, Thailand
A. aff. *nitens*	H203	-	FN252421	Phitsanulok Province, Thailand
A. aff. *nitens*	H215	-	FN252422	Phitsanulok Province, Thailand
A. aff. *nitens*	H254	-	FN252423	Phitsanulok Province, Thailand
A. aff. *nitens*	SUT081	-	DQ322105	Nakhon Ratchasima Province, Thailand
A. aff. *nitens*	SUT236	-	DQ322106	Trad Province, Thailand
A. aff. *nitens*	SUT242	-	DQ322107	Trad Province, Thailand
A. aff. *nitens*	SUT244	-	DQ322108	Trad Province, Thailand
A. aff. *nitens*	SUT251	-	DQ322109	Trad Province, Thailand

A. aff. *nitens*	SUT285	-	DQ322110	Kanchanaburi Province, Thailand
A. purpureonitens	H095	-	FM209442	Phitsanulok Province, Thailand
A. purpureonitens	H117	-	FM209448	Phitsanulok Province, Thailand
A. purpureonitens	H124	-	FN252416	Phitsanulok Province, Thailand
A. purpureonitens	H125	-	FN252417	Phitsanulok Province, Thailand
A. purpureonitens	H146	-	FM209451	Lumpang Province, Thailand
A. purpureonitens	H147	-	FM209452	Lumpang Province, Thailand
A. purpureonitens	SUT001	-	DQ322100	Nakhon Ratchasima Province, Thailand
A. purpureonitens	SUT004	-	DQ223753	Nakhon Ratchasima Province, Thailand
A. purpureonitens	SUT005	-	DQ322101	Nakhon Ratchasima Province, Thailand
A. purpureonitens	SUT167	-	DQ223754	Yasothorn Province, Thailand
A. purpureonitens	SUT262	-	DQ223755	Trad Province, Thailand
A. purpureonitens	ST2448	-	DQ223756	Royal Forest Department, Bangkok, Thailand
A. purpureonitens	ST2485	-	DQ223757	Royal Forest Department, Bangkok, Thailand
A. squamulosum	GB14	EF026139	-	Taiwan
A. stygium	GB62	FJ008986	-	China
A. stygium	GB63	EU272529	-	Andean paramo ecosystem, Colombia
A. stygium	GB64	EU272517	-	Andean paramo ecosystem, Colombia
A. stygium	GB115	AJ390409	-	USA
A. stygium	SUT058	-	DQ223760	Ratchaburi Province, Thailand
A. stygium	SUT243	-	DQ223761	Trad Province, Thailand
A. stygium	GB7	FJ205451	-	Taiwan
A. stygium var. *annulatum*	H164	-	FM209457	Chiang Rai Province, Thailand
A. truncatum	GB3	FJ478107	-	China
A. truncatum	GB93	AY909026	-	-
A. truncatum	GB99	AF201716	-	-
A. urceolatum	SUT098	-	DQ322103	Songkhla Province, Thailand
Annulohypoxylon sp.	H213	-	FN252426	Phitsanulok Province, Thailand
Annulohypoxylon sp.	H255	-	FN252427	Phitsanulok Province, Thailand
Annulohypoxylon sp.	SUT231	-	DQ322102	Trad Province, Thailand
Annulohypoxylon sp.	ST2332	-	DQ322098	Royal Forest Department, Bangkok, Thailand
Hypoxylon anthochroum	SUT233	-	QD201125	Trad Province, Thailand
H. anthochroum	SUT240	-	QD201126	Trad Province, Thailand
H. carneum	GB110	AM749926	-	Strain CBS 119310
H. cinnabarinum	GB125	AJ390398	-	USA
H. commutatum	H060	-	FM209434	Phitsanulok Province, Thailand
H. commutatum	H097	-	FM209443	Phitsanulok Province, Thailand
H. crocopeplum	H092	-	FM209441	Phitsanulok Province, Thailand
H. crocopeplum	H119	-	FN252428	Phitsanulok Province, Thailand
H. diatrypeoides	GB91	DQ985381	-	Mexico

H. diatrypeoides	GB92	DQ985380	-	Mexico
H. diatrypeoides	GB97	AY303629	-	Mexico
H. diatrypeoides	H221	-	FN252429	Phitsanulok Province, Thailand
H. diatrypeoides	H226	-	FN252430	Phitsanulok Province, Thailand
H. diatrypeoides	H235	-	FN252431	Phitsanulok Province, Thailand
H. duranii	H204	-	FN252432	Phitsanulok Province, Thailand
H. duranii	H238	-	FN252433	Phitsanulok Province, Thailand
H. duranii	H250	-	FN252434	Phitsanulok Province, Thailand
H. duranii	H253	-	FN252435	Phitsanulok Province, Thailand
H. duranii	H257	-	FN252436	Phitsanulok Province, Thailand
H. duranii	H261	-	FN252437	Phitsanulok Province, Thailand
H. fendleri	GB104	AJ390400	-	
H. fendleri	H078	-	FM209440	Phitsanulok Province, Thailand
H. fendleri	H111	-	FM209445	Phitsanulok Province, Thailand
H. fendleri	H128	-	FN252453	Phitsanulok Province, Thailand
H. fendleri	SUT061	-	QD201130	Ratchaburi Province, Thailand
H. fendleri	SUT120	-	QD201131	Petchaboon Province, Thailand
H. fendleri	SUT159	-	QD201132	Yasothorn Province, Thailand
H. fendleri	SUT162	-	QD223735	Yasothorn Province, Thailand
H. fendleri	SUT165	-	QD223736	Yasothorn Province, Thailand
H. fendleri	SUT280	-	QD223737	Kanchanaburi Province, Thailand
H. fragiforme	GB38	AY616690	-	-
H. fragiforme	GB39	AY616689	-	-
H. fragiforme	GB42	AY618235	-	Sweden
H. fragiforme	GB44	EU715619	-	Mexico
H. fragiforme	GB45	EU715613	-	Mexico
H. fragiforme	GB66	EF155530	-	Germany
H. fragiforme	GB67	EF155529	-	Germany
H. fragiforme	GB68	EF155528	-	Germany
H. fragiforme	GB69	EF155527	-	Germany
H. fragiforme	GB70	EF155526	-	Germany
H. fragiforme	GB71	EF155525	-	Germany
H. fragiforme	GB72	EF155524	-	Germany
H. fragiforme	GB73	EF155523	-	Germany
H. fragiforme	GB74	EF155522	-	Germany
H. fragiforme	GB75	EF155521	-	Germany
H. fragiforme	GB76	EF155520	-	Germany
H. fragiforme	GB77	EF155519	-	Germany
H. fragiforme	GB78	EF155518	-	Germany
H. fragiforme	GB79	EF155517	-	Germany
H. fragiforme	GB80	EF155516	-	Germany
H. fragiforme	GB81	EF155515	-	Germany
H. fragiforme	GB82	EF155514	-	Germany
H. fragiforme	GB83	EF155513	-	Germany
H. fragiforme	GB84	EF155512	-	Germany
H. fragiforme	GB85	EF155511	-	Germany
H. fragiforme	GB86	EF155510	-	Germany
H. fragiforme	GB87	EF155509	-	Germany
H. fragiforme	GB88	EF155508	-	Germany

H. fragiforme	GB89	EF155507	-	Germany
H. fragiforme	GB103	AF201709	-	-
H. fragiforme	GB121	AJ390403	-	USA
H. fragiforme	GB122	AJ390402	-	USA
H. fragiforme	GB123	AJ390401	-	USA
H. fragiforme	GB129	AJ246218	-	United Kingdom
H. fragiforme	GB130	AJ246217	-	USA
H. fragiforme	GB131	AJ246216	-	USA
H. fragiforme	GB132	AJ246215	-	USA
H. fuscopurpureum	GB96	AY945224	-	-
H. fuscum	GB26	AY616702	-	-
H. fuscum	GB27	AY616701	-	-
H. fuscum	GB28	AY616700	-	-
H. fuscum	GB29	AY616699	-	-
H. fuscum	GB30	AY616698	-	-
H. fuscum	GB31	AY616697	-	-
H. fuscum	GB32	AY616696	-	-
H. fuscum	GB33	AY616695	-	-
H. fuscum	GB34	AY616694	-	-
H. fuscum	GB35	AY616693	-	-
H. fuscum	GB100	AF201715	-	Strain ATCC36663
H. fuscum	GB119	AJ390405	-	USA
H. fuscum	GB120	AJ390404	-	USA
H. haematostroma	GB111	AM749924	-	Strain MUCL 47600
H. haematostroma	H065	-	FM209436	Phitsanulok Province, Thailand
H. haematostroma	H105	-	FN252438	Phitsanulok Province, Thailand
H. haematostroma	H108	-	FN252439	Phitsanulok Province, Thailand
H. haematostroma	H109	-	FM209444	Phitsanulok Province, Thailand
H. haematostroma	H114	-	FM209446	Phitsanulok Province, Thailand
H. haematostroma	H115	-	FN252440	Phitsanulok Province, Thailand
H. haematostroma	H142	-	FM209450	Lumpang Province, Thailand
H. haematostroma	H264	-	FM209466	Phitsanulok Province, Thailand
H. haematostroma	H276	-	FN252441	Phitsanulok Province, Thailand
H. haematostroma	SUT164	-	QD223738	Yasothorn Province, Thailand
H. haematostroma	SUT292	-	QD223739	Kanchanaburi Province, Thailand
H. haematostroma	SUT293	-	QD223740	Kanchanaburi Province, Thailand
H. howeanum	GB109	AM749928	-	Strain MUCL 47599
H. hypomiltum	SUT166	-	QD322114	Yasothorn Province, Thailand
H. intermedium	GB127	AJ390396	-	-
H. investiens	H259	-	FN252442	Phitsanulok Province, Thailand
H. investiens	SUT041	-	QD322115	Ratchaburi Province, Thailand
H. investiens	SUT063	-	QD322116	Ratchaburi Province, Thailand
H. investiens	GB4	FJ185308	-	Strain CBS 118185; New Zealand
H. investiens	GB5	FJ185307	-	Strain CBS 118183; Malaysia
H. kanchanapisekii	SUT066	-	QD223741	Ratchaburi Province, Thailand
H. kanchanapisekii	SUT068	-	QD223742	Ratchaburi Province, Thailand
H. kanchanapisekii	SUT069	-	QD223743	Ratchaburi Province, Thailand
H. lenormandii	H063	-	FM209435	Phitsanulok Province, Thailand
H. lenormandii	H212	-	FM209463	Phitsanulok Province, Thailand

H. lenormandii	H217	-	FN252456	Phitsanulok Province, Thailand
H. lenormandii	SUT016	-	QD223744	Burirum Province, Thailand
H. lenormandii	SUT180	-	QD223745	Nakhon Ratchasima Province, Thailand
H. lenormandii	ST2324	-	QD223746	Royal Forest Department, Bangkok, Thailand
H. lividipigmentum	H075	-	FM209438	Phitsanulok Province, Thailand
H. lividipigmentum	H173	-	FN252454	Phitsanulok Province, Thailand
H. lividipigmentum	H208	-	FN252455	Phitsanulok Province, Thailand
H. macrocarpum	GB23	AY616705	-	-
H. macrocarpum	GB24	AY616704	-	-
H. macrocarpum	GB25	AY616703	-	-
H. macrocarpum	H245	-	FN252444	Phitsanulok Province, Thailand
H. monticulosum	GB17	DQ631938	-	Bahn Pha Deng, Chiang Mai Province, Thailand
H. monticulosum	H076	-	FM209439	Phitsanulok Province, Thailand
H. monticulosum	H155	-	FM209454	Chiang Rai Province, Thailand
H. monticulosum	H188	-	FM209458	Phitsanulok Province, Thailand
H. monticulosum	H206	-	FM209462	Phitsanulok Province, Thailand
H. monticulosum	H247	-	FN252443	Ratchaburi Province, Thailand
H. monticulosum	H272			Phitsanulok Province, Thailand
H. monticulosum	SUT042	-	QD223747	Nakhon Ratchasima Province, Thailand
H. monticulosum	SUT080	-	QD223748	Songkhla Province, Thailand
H. monticulosum	SUT116	-	QD223749	-
H. nicaraguense	GB113	AM749922	-	Strain CBS 117739
H. notatum	H234	-	FN252445	Phitsanulok Province, Thailand
H. notatum	H268	-	FN252446	Phitsanulok Province, Thailand
H. ochraceum	GB118	AJ390406	-	USA
H. papillatum	GB102	AF201710	-	-
H. pelliculosum	H227	-	FN252447	-
H. pelliculosum	H233	-	FN252448	-
H. perforatum	GB61	FJ464593	-	China
H. perforatum	GB107	AM749935	-	-
H. perforatum	GB117	AJ390407	-	USA
H. perforatum	SUT020	-	QD322118	Burirum Province, Thailand
H. perforatum	SUT218	-	QD322119	Trad Province, Thailand
H. perforatum	SUT294	-	QD322120	Kanchanaburi Province, Thailand
H. petriniae	GB112	AM749923	-	Strain CBS 115158
H. pilgerianum	H064	-	FM209437	Phitsanulok Province, Thailand
H. polyporus	GB106	AM749941	-	-
H. retpela	H116	-	FM209447	Phitsanulok Province, Thailand
H. rickii	GB116	AJ390408	-	USA
H. rogersii	GB90	DQ983229	-	Maulan, Libo County, Guizhou Province, China
H. rubigineoareolatum	H110	-	FN252450	Phitsanulok Province, Thailand
H. rubiginosum	GB9	AY787708	-	Lithuania
H. rubiginosum	GB10	EF026143	-	United Kingdom
H. rubiginosum	GB108	AM749936	-	Strain CBS 119309

H. rubiginosum	SUT215	-	QD223758	Trad Province, Thailand
H. rubiginosum	SUT221	-	QD223759	Trad Province, Thailand
H. rubiginosum var. *microsporum*	H229	-	FN252449	Phitsanulok Province, Thailand
H. shearii var. *minor*	GB11	EF026142	-	Mexico
H. subdisciforme	GB8	FJ172269	-	Strain ATCC MYA-4107
H. subgilvum var. *microsporum*	SUT104	-	QD322121	Songkhla Province, Thailand
H. subgilvum var. *microsporum*	SUT108	-	QD322122	Songkhla Province, Thailand
H. sublenormandii	H237	-	FN252451	Phitsanulok Province, Thailand
H. sublenormandii	SUT250	-	QD223762	Trad Province, Thailand
H. sublenormandii	SUT282	-	QD223763	Kanchanaburi Province, Thailand
H. subrutiloides	GB6	FJ185304	-	Strain F202416; New Zealand
H. suranareei	H224	-	FN252452	Phitsanulok Province, Thailand
H. suranareei	SUT182	-	QD223764	Nakhon Ratchasima Province, Thailand
H. suranareei	SUT183	-	QD223765	Nakhon Ratchasima Province, Thailand
Nemania serpens	GB2	HM036598	-	Lithuania
Nemania serpens	GB18	AY781226	-	Sweden
Nemania serpens	GB19	AY805565	-	Sweden
Nemania serpens	GB46	FJ903321	-	-

Table S2: The NCBI BLASTN Default Scoring System.

The accompanying gap penalty is $g(i) = 5 + 2i$ for a gap of length i.

Any aligned letter-pair containing an ambiguous nucleotide was given score 0.

	A	C	G	T
A	1	-2	-2	-2
C	-2	1	-2	-2
G	-2	-2	1	-2
T	-2	-2	-2	1

Table S3: The UCSC BLASTZ Default Scoring System.

The accompanying gap penalty is $g(i) = 400 + 30i$ for a gap of length i.

Any aligned letter-pair containing an ambiguous nucleotide was given score 0.

	A	C	G	T
A	91	-114	-31	-123
C	-114	100	-125	-31
G	-31	-125	100	-114
T	-123	-31	-114	91

Table S4: Probability of Correct Identification (PCI) by Species.

The PCI uses multiple alignment and p-distance. Note: in *Annulohypoxylon cohaerens*, ITS analysis correctly identified only 0.25 of the samples, so the species as a whole is not correctly identified.

Species	PCI
Annulohypoxylon annulatum	0
Annulohypoxylon archeri	0
Annulohypoxylon atroroseum	0
Annulohypoxylon bovei	1
Annulohypoxylon cohaerens	0.25
Annulohypoxylon moriforme	0
Annulohypoxylon multiforme	0
Annulohypoxylon nitens	1
Annulohypoxylon aff. *nitens*	1
Annulohypoxylon purpureonitens	1
Annulohypoxylon stygium	0
Annulohypoxylon truncatum	0
Hypoxylon anthochroum	0
Hypoxylon commutatum	0
Hypoxylon crocopeplum	0
Hypoxylon diatrypeoides	0
Hypoxylon duranii	1
Hypoxylon fendleri	0
Hypoxylon fragiforme	0
Hypoxylon fuscum	1
Hypoxylon haematostroma	1
Hypoxylon investiens	0
Hypoxylon kanchanapisekii	1
Hypoxylon lenormandii	0
Hypoxylon lividipigmentum	0
Hypoxylon macrocarpum	0
Hypoxylon monticulosum	1
Hypoxylon notatum	1
Hypoxylon pelliculosum	1
Hypoxylon perforatum	0
Hypoxylon rubiginosum	0
Hypoxylon subgilvum	0
Hypoxylon sublenormandii	1
Hypoxylon suranareei	1
Nemania serpens	0

References

1. Hayashi K, Hatsutori N, Donho M, Nakajima H (1996) Dihydrofolate reductase inhibitors containing mitorubrin compounds for protozoiasis. Japanese Patent 08217673.
2. Ju Y-M, Rogers JD (1996) A revision of the genus *Hypoxylon*. Mycologia Mem 20: 1-365.
3. Ju Y-M, Rogers JD (2002) The genus *Nemania* (Xylariaceae). Nova Hedwigia 74: 75-120.
4. Miller JH (1961) A monograph of the world species of Hypoxylon. Athens, U.S.A.: University of Georgia Press.
5. Rayner RW (1970) A mycological colour chart. Commonwealth Mycological Institute, Kew.
6. O'Donnell K, Cigelnik E (1997) Two divergent intragenomic rDNA ITS2 types within a monophyletic lineage of the fungus *Fusarium* are nonorthologous. Mol Phylogenetics Evol 7: 103–116.
7. Hsieh HM, Ju YM, Rogers JD (2005) Molecular phylogeny of *Hypoxylon* and closely related genera. Mycologia 97: 844-865.
